# Chia Seed Mucilage Edible Films with *Origanum vulgare* and *Satureja montana* Essential Oils: Characterization and Antifungal Properties

**DOI:** 10.3390/membranes12020213

**Published:** 2022-02-11

**Authors:** Nuria Muñoz-Tébar, Manuel Carmona, Gonzalo Ortiz de Elguea-Culebras, Ana Molina, María Isabel Berruga

**Affiliations:** 1 Food Quality Research Group, Institute for Regional Development (IDR), Universidad de Castilla-La Mancha, 02071 Albacete, Spain; nuria.munoz@uclm.es (N.M.-T.); manuel.carmona@uclm.es (M.C.); ana.molina@uclm.es (A.M.); 2 Centro de Investigación Agroforestal de Albaladejito (IRIAF-JCCM), Carretera Toledo-Cuenca km 174, 16194 Cuenca, Spain; gonzaloo@jccm.es

**Keywords:** chia by-products, chia mucilage films, essential oils, antifungal activity, bioactive films

## Abstract

Films made with mucilage obtained from defatted chia seeds and incorporated with oregano (*Origanum vulgare*) and savory (*Satureja montana*) essential oils (0.1,1.0 and 1.5% *v*/*v*) were prepared to evaluate their physical, optical, mechanical and antifungal properties as well as their microstructure. The use of different types of essential oils (oregano or savory) only had a significant effect on the light transmittance, total color difference (∆E) and antifungal activity of the films. However, the kind of essential oil was not significant for the physical, optical and mechanical properties of the films. Increasing concentrations of essential oils up to 1.5% *v*/*v* led to a decrease in tensile strength (TS) and elongation at break (EB). Antifungal properties significantly increased with the incorporation of essential oils (*p* < 0.05). The antifungal activity of the chia mucilage films incorporated with *O. vulgare* and *S. montana* essential oil was screened by agar disc-diffusion assay against five mold strains commonly found in foods. Films containing 0.1% *v*/*v* of essential oils were not active, whereas films containing 1.0 and 1.5% *v*/*v* were very effective at inhibiting the growth of the tested mold strains (38.01–77.66%). Scanning electron microscopy showed that incorporation of essential oils caused some heterogeneity in the films and the surface displayed no pores or cracks as well as a better integration of oregano EO in the polymeric network. The results pointed out that the incorporation of oregano and savory essential oils as a natural antimicrobial agent has appreciable potential for the development of films as active packaging to control mold contamination and increase food safety.

## 1. Introduction

The growing consumption of synthetic plastic of petroleum-derived materials along with the slow degradation and the problems of recycling these materials has caused a large accumulation in the environment during the last decades. This is leading to a serious pollution problem due to its non-biodegradability [1]. The raising environmental concerns of consumers regarding the harmful effects of plastic packaging and coatings has led to research on packaging focused on biodegradable films made from renewable and eco-friendly sources as a suitable substitute for conventional plastics [2,3]. In response to this, the researchers and agri-food industry are looking for alternative materials for food coating consisting of natural, safe, renewable and recyclable ingredients that increase their biodegradability, biocompatibility and edibility [4]. Bio-based polymers, such as polysaccharides, lipids, proteins or a combination of them, are the most common and promising materials for the development of biodegradable films and coatings [2,3]. Among the edible films made from polysaccharides, the most frequently used raw materials in the literature are chitosan, starch and gums, and the studies about the use of mucilage are still limited. However, the technological properties and dietary characteristics of mucilage obtained from plant seeds have led them to be regarded as one of the most promising materials in the development of edible coatings and films [5]. According to the literature, the seeds used so far for the development of films and coatings from mucilage incorporated with essential oils are balangu seed [6], basil seed [7], chia seed [8], cress seeds [9] and quince seeds [10,11], but in comparison with other materials, very little work has been carried out; therefore it is a field of research with considerable potential that should be studied more and in which the present work can provide valuable information for future work.

The agri-food industry generates large amounts of wastes, such as seeds, peels or defatted flours, and agricultural by-products are rich sources of polysaccharides with film-forming properties [12,13]. In this context, some of the seeds that produce mucilage also contain a significant amount of omega 3, as is the case of chia seeds, and therefore are mainly used to obtain oils rich in this essential fatty acid. This generates a large amount of by-products, such as defatted seeds, that once grounded can also be used to obtain mucilage for the production of edible and biodegradable films and coatings as demonstrated in our previous work [12].

Chia mucilage consists of a branched matrix of xylose, glucose and glucuronic acid [14], and has been described as a new potential source of polysaccharide gum by the FAO (Food Agriculture Organization) due to its mucilaginous properties at low concentrations in aqueous solutions [15]. Moreover, edible and biodegradable films and coatings can act as a carriers of bioactive compounds, such as antioxidants and antimicrobial agents [3,16,17,18,19], to act against the microbial spoilage and deterioration of food [20]. Antimicrobial films and coatings are gaining greater attention from food and packaging industries due to the increasing consumer demand for natural, minimally processed and synthetic preservative-free food products [19,21]. Therefore, special attention has been driven to applying essential oils (EOs) as potential sources of bioactive compounds that do not represent a risk to the health of the consumer [22]. EOs extracted from plants are natural antimicrobial agents with long-recognized antimicrobial properties against a wide range of microorganisms [23] as well as being classified as GRAS (generally recognized as safe) [24]. Essential oils are usually incorporated into the films to improve their mechanical barrier properties as well as to provide them with antimicrobial capacity [16,25]. Among a great variety of essential oils, oregano EO (OEO), extracted from *Origanum vulgare*, is widely used for its antioxidant and antimicrobial activity [26,27]. The strong antimicrobial capacity of this EO and other essential oils, such as the savory essential oil (SEO) extracted from *Satureja montana*, is mainly due to that carvacrol is their main constituent [28]. Even though antimicrobial films incorporated with oregano EO have been evaluated by several studies [7,29], limited data exist on the incorporation of savory EO in edible and biodegradable films.

The aim of this study was to incorporate savory or oregano essential oils into chia by-product edible films and characterize the effect of the EOs’ addition in the physical, optical, mechanical and barrier properties of the films as well as to evaluate the in vitro antifungal activity of the films against selected molds isolated from pressed sheep cheese.

## 2. Materials and Methods

### 2.1. Materials

Commercial organic chia seeds (Biogran S.L., Madrid, Spain) were purchased and had a mean composition (g/100 g): 31.1 of fat, 21.2 of protein, 3.8 of saturated fatty acids (SFAs) and 17.8 of α-linolenic acid. Glycerol (analytical grade) and Tween 20 (food grade) were obtained from Guinama S.L.U. (Valencia, Spain) and Sigma–Aldrich (St. Louis, MO, USA), respectively. *Satureja montana* and *Origanum vulgare* organic plants were purchased from Josenea Bio (Navarra, Spain) and the essential oils were obtained by solvent-free steam distillation and analyzed by GC-FID following the methods described by Muñoz-Tebar et al. [30]. The main constituents of savory EO were carvacrol (77.59%), p-cymene (6.62%), isoborneol (2.23%), γ-terpinene (1.28%) and linalool (1.23%), and the major compounds of oregano EO were carvacrol (82.41%), thymol (4.98%), trans-caryophyllene (2.70%), p-cymene (2.43%) and γ-terpinene (1.82%).

### 2.2. Defatting of Chia Seeds and Mucilage Extraction

Chia seeds were defatted in a hydraulic press (MECAMAQ Model DEVF 80, Vila-Sana, Lleida, Spain) as described by Muñoz-Tebar et al. [31]. Chia mucilage (CM) was extracted from defatted seeds following the procedure described by Muñoz-Tebar et al. [12]. Briefly, 10 g of defatted chia seeds were extracted with distilled water (pH previously adjusted to 8 with 0.1 M NaOH) at a ratio 1:40 (flour: water) during 2 h at 80 ± 2 °C under constant stirring. The solution was then centrifuged (Beckman Ultracentrifuge, Pasadena, CA, USA) at 10,000 rpm for 30 min at 20 °C, filtered through a gauze, spread on a tray and dried at 50 °C overnight. Finally, the dried mucilage was recovered and stored in a desiccator until use.

### 2.3. Production of Chia Mucilage Films Incorporated with Essential Oils

Three sets of chia mucilage films were prepared using the same method as Muñoz-Tebar et al. [12] and the essential oil was incorporated following the procedure described by Jouki et al. [11] with slight modifications. Briefly, 1.5% *w*/*v* of CM was dissolved in distilled water (pH was adjusted to 9 with 0.1 M NaOH) stirred at 25 °C for 3 h and heated at 80 °C during 30 min under stirring. Subsequently, 35% of glycerol and 15% of Tween 20 (*w*/*w* based on CM weight) were added and the film solution was stirred 30 min at room temperature. After that, the corresponding concentration of each EO (0.1, 1.0 and 1.5% *v*/*v*) was added, the solution was homogenized at 12,000 rpm for 5 min in a homogenizer (Ultra-Turrax T25 BASIC, IKA-Werke GmbH & Co. KG, Staufen, Germany) and placed in an ultrasonic bath during 2 min to remove air bubbles. Finally, the film solutions at 0.55 g/cm^2^ were casted onto Petri dishes of 55, 90 and 140 mm ø, placed in an oven with air convection (Heraeus, Hanau, Germany) dried at 35 °C overnight and stored in a desiccator at 25 °C and 52% RH for at least 48 h prior to their characterization.

### 2.4. Characterization of Chia Mucilage EO Films

#### 2.4.1. Moisture Content and Water Solubility (WS)

The determination of moisture content and water solubility of the films was carried out according to Dick et al. [15] with slight modifications. For moisture, film samples of 2 cm diameter were dried at 105 °C in an oven (J.P. Selecta, Barcelona, Spain) and the moisture content was gravimetrically calculated in triplicate after 24 h of drying. The water solubility of the films was determined in triplicate with the dried films from the moisture analysis using the following equation:

WS (%) = [(W_i_ − W_f_)/W_i_] × 100 (1)

being W_i_ the initial dry weight and W_f_ the weight resulting of the dried material.

#### 2.4.2. Water Vapor Permeability (WVP)

The water vapor permeability of the films were determined in triplicate based on Muñoz-Tebar et al. [12] and the ASTM E96/E96M-16 standard [32]. Chia films circles (55 mm diameter) were manually sealed sticking on the top of the permeation cells (1.22 × 10^−2^ m^2^) containing 50 mL of distilled water, placed inside a desiccator with silica gel (0% HR, 24 °C) and weighed during 10 h at 2 h intervals following the schedule used by other authors [33,34,35].

Finally, water vapor permeability (WVP) of chia films was calculated with the following equation:

(2) $$WVP\;\left(g\;s^{-1}m^{-1}\;Pa^{-1}\right)=\frac{w\;\times L}{A\;\times \;t\;\times \Delta P}$$

where w is the weight of the water that permeated through the film (g), L is the film thickness (m), A is the permeation area of the cell (m^2^), t is the time of permeation (s) and ΔP is the water vapor partial difference (Pa) across the two sides of the film.

#### 2.4.3. Color and Opacity

The color (coordinates CIEL*a*b*) of the films was measured in the reflection mode with a Minolta CR-400 colorimeter (Minolta, Japan) equipped with a CR-A33a cone, a D65 illuminant and an angle vision of 10 degrees following the method described by Costa et al. [33]. For calibration, a white standard color plate (L* = 97.24, a* = 0.09 and b* = 1.86) was used and the L*, a* and b* coordinates were measured in triplicate. The opacity of the films was calculated in triplicate according to the Hunter Lab method [33] with the following equation:

Opacity (%) = [Y_b_ − Y_w_)/Y_b_] × 100 (3)

where Y_b_ is the opacity on a black standard and Y_w_ is the opacity on a white standard.

The total color difference (∆E) was calculated according to Dick et al. [15] with a white standard plate (L* = 97.24, a* = 0.09 and b* = 1.86).

#### 2.4.4. Light Transmittance

The capacity of light transmittance of chia films was measured according to Dick et al. [15] with minor modifications. Chia film samples (4 cm × 0.8 cm) were placed in a spectrophotometer cell and transmittance was measured in triplicate by spectrum scanning (wavelengths from 200 to 900 nm) with a spectrophotometer UV-Vis GENESYS 150 (Thermo Fisher Scientific, Waltham, MA, USA). Air was used as reference and the transmittance values (expressed as % of transmittance) were measured in triplicate.

#### 2.4.5. Thickness and Mechanical Properties

Thickness of the films (mm) was measured in triplicate at three different points for each sample using a digital micrometer IP65 Coolant-Proof (Mitutoyo, Japan) with a precision of ±0.01 mm. Mechanical properties of chia films were determined according to the procedure described in the standard ASTM D882-10 [36] in a texturometer TA-XT2i (Stable Micro Systems Ltd., Godalming, Surrey, UK). For that, films (2 cm × 10 cm) were placed between the A/MTG tensile grips with an initial distance of 80 mm and the force and deformation were recorded at a speed of 0.00083 m/s. Nine strips from each film were measured and tensile strength (TS) and elongation at break (EB) were expressed in MPa and % elongation, respectively.

### 2.5. Antifungal Capacity

The in vitro antifungal activity of the films was carried out according to Sapper et al. [19] with minor modifications. Stock cultures of *Aspergillus flavus* CECT 2687, *Penicillium verrucosum* CECT 2906 supplied by the Spanish Type Culture Collection (CECT, Burjassot, Spain) as well as 3 wild strains isolated from pressed sheep cheese and identified as *Penicillium crustosum* 1A01, *Aspergillus puulaauensis* 1A05 and *Penicillium commune* 301 in a previous study [30]. The molds were cultivated and incubated in Agar Potato Dextrose (PDA, Merck, Darmstadt, Germany) at 25 °C until sporulation and used after 7 days of active growth. Films antifungal capacity against the selected molds was evaluated by spreading 100 µL of each inoculum (1–2 × 10^6^ cfu/mL) in PDA dishes. Then, film discs of 10 mm diameter were placed on the center of the Petri dishes. Later, the plates were incubated at 25 °C for 3 days and the mold growth was evaluated by measuring the diameter of the inhibition halos (4 measurements for each plate) in millimeters using the software ImageJ v1.52a. The experiment was performed in duplicate and the % of growth inhibition was calculated using the following equation:

% Growth inhibition = [A_c_ − A_e_)/A_c_] × 100 (4)

where A_c_ is the growth zone of the control plate (without EO) and A_e_ is the growth zone of the plates containing the EO-film discs.

### 2.6. Scanning Electron Microscopy (SEM)

Films were cut in pieces of 0.5 × 0.5 cm^2^ [37] and then samples were coated with Au-Pt using an SC7620 sputter coater by EMITECH to avoid surface charging-up problems and improve the final image resolution. After that, samples were placed on the sample holder and the SEM micrographs were recorded on a 6490LV JEOL electron microscope operating at 20 kV.

### 2.7. Statistical Analysis

Statistical analysis of data was performed using SPSS (IBM SPSS Statistics version 26). ANOVA (one way) was calculated using a confidence level of 95% to determine any significant difference between the films containing the two essential oils and the different concentrations tested. When there was a significant difference (*p* < 0.05), Tukey test was carried out to determine the differences between films made with different concentrations (0.0, 0.1, 1.0 and 1.5% *v*/*v*) of oregano and savory essential oils.

## 3. Results and Discussion

### 3.1. Moisture Content, Water Solubility, Thickness and Water Vapor Permeability

The results of the moisture analysis of the films made with different concentrations of oregano and savory EOs are presented in Table 1. No significant differences were noticed due to the use of different essential oils in the film formulation. Regarding the effect of EO incorporation into chia mucilage-based films, a significant increase in moisture was observed at the highest concentration (5 and 8% in the films containing oregano and savory EOs, respectively). As Table 1 shows, increasing EO concentrations led to a significant increase in the moisture content, ranging from 23.31 to 27.69% in the films formulated with oregano and from 25.73 to 30.83% in films embedded with savory essential oil.

An increase in moisture content (from 20.97 to 28.56%) due to the addition of EO was also observed by Go and Song [38] when java citronella EO was incorporated into *Gelidium corneum*-chitosan films at concentrations of 0.5, 1.0 and 1.5% *v*/*v*. Hosseini et al. [39] and Jouki et al. [10] reported that this increase could be explained due to the amount of water molecules presented in the polymer chains and the loosening of the film matrix.

Moreover, it was noticed that the moisture of films containing EOs were comparable to those obtained by other authors for chitosan-based films [38,39] and higher than the ones obtained by Jouki et al. [11] in films made with quince seeds and oregano EO, which ranged from 18.67 to 19.31%.

The solubility of films made only with chia mucilage was affected by the incorporation of both types of essential oils since an increase in solubility was observed at all concentrations studied (reaching values up to 11% higher at the highest concentration). Likewise, the results of water solubility of the films showed that the incorporation of different essential oils (oregano and savory) did not significantly (*p* > 0.05) affect the solubility of the films (Table 1).

Regarding the effect of increasing EO concentration in the films, a significant rise in WS was observed when the highest concentration was used (Table 1). Aguirre et al. [40] stated that incorporation of essential oils into films could cause an increase in soluble matter, thus leading to films with higher WS due to a lower density polymeric network caused by similar structures between the glycerol and the phenolic compounds present in the EOs. Overall, it was observed that water solubility of the chia mucilage essential oil films was higher than those obtained in chitosan-based films with java citronella EO (28.16% at 1.5% *v*/*v*) [38], triticale protein films (46.48% at 2% *v*/*v*) [40], quince seed films (48.85% at 1.5% *v*/*v*) [11] and fish gelatin–chitosan films (54.76% at 1.2% *v*/*v*) [3] containing different concentrations of oregano essential oil. The adequate solubility of the films will depend on their application or intended use; therefore, the high solubility indicates that the films would not be suitable for the protection of food with high water activity as they are highly biodegradable when they come into contact with water.

The base film made with mucilage extracted from defatted chia seeds had a thickness of 0.123 mm [12], so the incorporation of essential oils caused a significant decrease in film thickness in all concentrations studied. However, it was observed that the use of different types of essential oils did not influence the thickness (Table 1). Thickness of the films with essential oils varied from 0.075 to 0.096 mm and considering the effect of increasing EO concentration, no significant differences (*p* > 0.05) were noticed, as found by Gahruie et al. [17] when they incorporated *Zataria multiflora* EO in basil seed gum-based edible films.

Thickness of films embedded with EOs was comparable to those obtained by Nisar et al. [41] in pectin films incorporated with clove oil (0.094 mm at 1.5% *v*/*v*) and higher than those obtained for quince seed films with oregano EO (0.079 mm at 2% *v*/*v*) [11] and carrageenan films containing *Satureja hortensis* EO (0.068 mm at 3% *v*/*v*) [42]. The differences in thickness found in the literature may be due to the different methods, matrices and concentrations of EOs used in the film formulation.

The water vapor permeability (WVP) is another relevant property to consider in the development of films as it regulates water transfer between the film and the external environment [43]; therefore, it should be as low as possible to prevent excessive water diffusion through the film [19]. In general, an enhancement in water barrier properties was observed with the addition of EO (both oregano and savory) compared to previously reported WVP values in films made only with mucilage from defatted chia seeds (0.58 × 10^−10^ g/s m Pa, [12]). As Table 1 shows, no significant differences were found in the water vapor permeability due to the used of different essential oils or increasing concentrations. When comparing the results to other films, it was observed that the WVP values achieved in the present study were lower than those addressed by Pelissari et al. [29] in cassava starch–chitosan with oregano EO (0.62 × 10^−10^ g/s m Pa at 1.0%) by Hashemi et al. [7] in basil-seed films with oregano EO (0.41 × 10^−10^ g/s m Pa at 2%) and by Jouki et al. [10,11] in quince seeds containing oregano EO (1.1 × 10^−10^ g/s m Pa at 1.5%) and thyme EO (0.99 × 10^−10^ g/s m Pa at 1.5%). Improving the water barrier properties of films is one of the major problems to be faced in the development of new films for food applications [3] and thus the values obtained in the present work could indicate that these films would be a better barrier to water vapor than other biodegradable and edible films found in the literature.

### 3.2. Color and Opacity

The main function of color in packaging is to draw consumers’ attention and influence their product perception and purchase decisions [44]. Another property that plays an important role in the development of new films is the opacity, which is closely related to the appearance of the coated food and its commercial value as well as determine their adequacy for several applications [45]. Therefore, the effect of the EOs’ addition on color coordinates (CIEL*a*b*), total color difference (ΔE) and opacity of the films were evaluated in the films studied (Table 2). The type of essential oil used (*O. vulgare* or *S. montana*) only had a significant effect (*p* < 0.01) on the total color difference (ΔE), being higher at all concentrations when savory EO was used.

In relation to the incorporation of essential oil at increasing concentrations, no significant differences were observed, neither in the L* and b* coordinates nor in the total color difference. However, a significant increase (*p* < 0.05) in the a* coordinate was observed when the essential oils were added at concentrations ranging from 0.1 to 1.5% *v*/*v*, so it could be said that the films tended to be more reddish (Table 2). The findings are consistent with Jouki et al. [11] who also observed an increase in coordinate a* as a result of the incorporation of oregano EO (1.0, 1.5 and 2.0% *v*/*v*) into quince seed films.

Moreover, opacity increased significantly in films formulated with 1.5% *v*/*v*, reaching values up to 5% higher than films without essential oil, and this fact has been previously observed by other authors when they added essential oils into biodegradable films [37,46].

These results demonstrate that increases up to 1.5% *v*/*v* of oregano and savory EO concentrations will not affect the color properties or opacity of the films. Martucci et al. [47] did not observe significant changes in color coordinates up to 3000 mg/kg of oregano EO in gelatin films and an increase of oregano and thyme EOs (from 0.5 to 2.0% *v*/*v*) did not affect the color coordinates of quinoa flour films developed by Pagno et al. [48].

### 3.3. Transmittance

**Figure 1:**
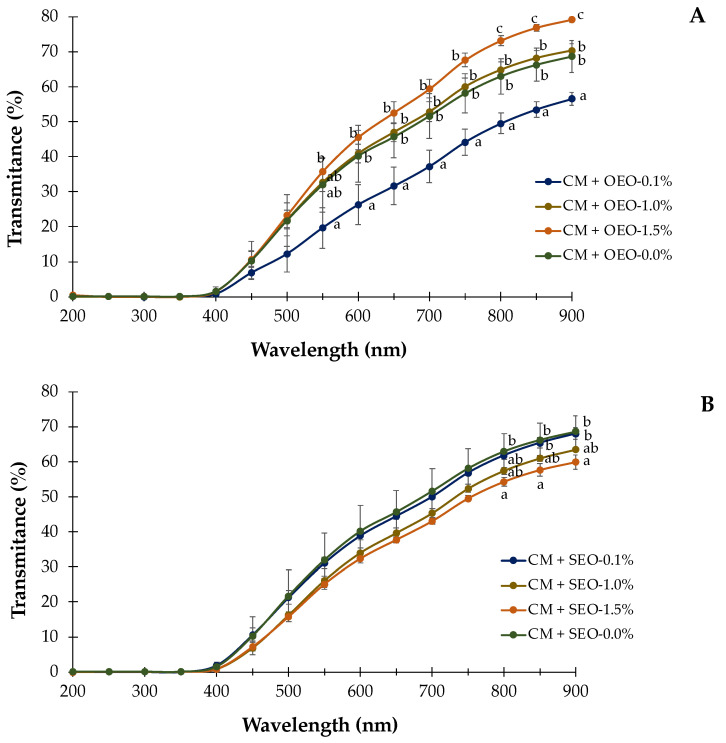
Effect of oregano (OEO; (**A**)) or savory (SEO; (**B**)) essential oil in the light transmittance (%) of chia mucilage films (CM, Muñoz-Tebar et al. [12]). ^a–c^ Different letter means significant differences (*p* < 0.05) due to EO concentration. Data without any superscripts mean that there were no significant differences (*p* > 0.05) either by the EO concentration effect (indicated with lower case letters) or by the EOs used in the formulation (indicated with capital letters).

Figure 1 represents the effect of using different essential oils on the light transmittance as well as the effect of increasing the EO concentration in the films. Capacity of light transmittance is one of the most important properties in the development of new films and packaging materials as it will allow us to identify the protection that the film will exert to the coated food when exposed to visible and UV light.

Considering the influence of the use of different EOs on the transmittance of the films, it was observed that there were no significant differences in the UV range (200–450 nm) and values of % transmittance were very low (from 0.03 to 1.92). This fact confirms that the use of these essential oils (oregano and savory) will not affect the UV light barrier properties of the films and that both essential oils would be suitable options for the development of films with protective properties against UV radiation. Likewise, it was noticed that the incorporation of EOs in the films (regardless of concentration) did not modify the UV barrier properties with respect to the values for chia mucilage-based edible films without essential oil.

On the contrary, the use of different essential oils affected the visible light barrier properties of the films since % transmittance values were lower in the films containing savory than in those containing oregano (Supplementary Table S1).

The incorporation of *S. montana* essential oil up to 1.5% *v*/*v* will improve the visible light barrier properties by reducing transmittance up to 24% at the highest concentration (59.93 vs. 79.19%), compared to the films containing *O. vulgare* essential oil. This characteristic could delay and prevent the reactions triggered by the action of visible light, such as lipid oxidation, loss of nutrients and discoloration or formation of off-flavors in foods [37].

Regarding the effect of the increasing EO concentrations, there were no significant differences (*p* > 0.05) in the films in the UV spectrum (from 200 to 450 nm) but significant differences were observed in the visible zone (from 500 to 900 nm in films containing oregano and from 800 to 900 m in films incorporated with savory). Figure 1 shows two opposite behaviors: the increase in oregano EO concentration (Figure 1A) caused a significant increase in the % transmittance reaching values of 79.19% at 1.5% *v*/*v*. This increase in transmittance could be due to its thymol content, which is not present in savory essential oil, as other authors noticed that the presence of thymol in starch films may block or decrease the intensity of light scattering causing an increase in transmittance [49]. However, increasing concentrations of savory EO (Figure 1B) enhanced the light transmittance capacity by decreasing the transmittance values (from 68.02% to 59.93%). The decrease in transmittance may be related to light scattering and lipid droplets dispersed in the film matrix that interfere with the light transmission [50]. Improvements in the rate of light transmittance have been previously reported by Ocak [50] in collagen hydrolysate films formulated with thyme EO (from 63.28% to 56.35%), in films made from kefiran, carboxymethyl cellulose and *Satureja khuzestanica* EO (from 84.09% to 36.78%) [37] and in *Gelidium corneum*-chitosan films with java citronella EO [38].

### 3.4. Mechanical Properties

Tensile strength (TS) and elongation at break (EB) were evaluated in order to describe the behavior of the films with EOs at increasing concentrations since the interaction between the polysaccharides from chia mucilage and the components of the essential oils will play an important role in these parameters. Moreover, the analysis of these parameters will help to better understand the mechanical characteristics of the films for food applications [42]. Comparing the results with the mechanical properties of the chia mucilage-based films (without EOs), it was found that the incorporation of *O. vulgare* and *S. montana* essential oils at 0.1% *v*/*v* enhanced the mechanical properties of the base films (containing only mucilage from defatted chia seeds) by increasing tensile strength and elongation at break up to 44% and 19%, respectively [12].

**Figure 2:**
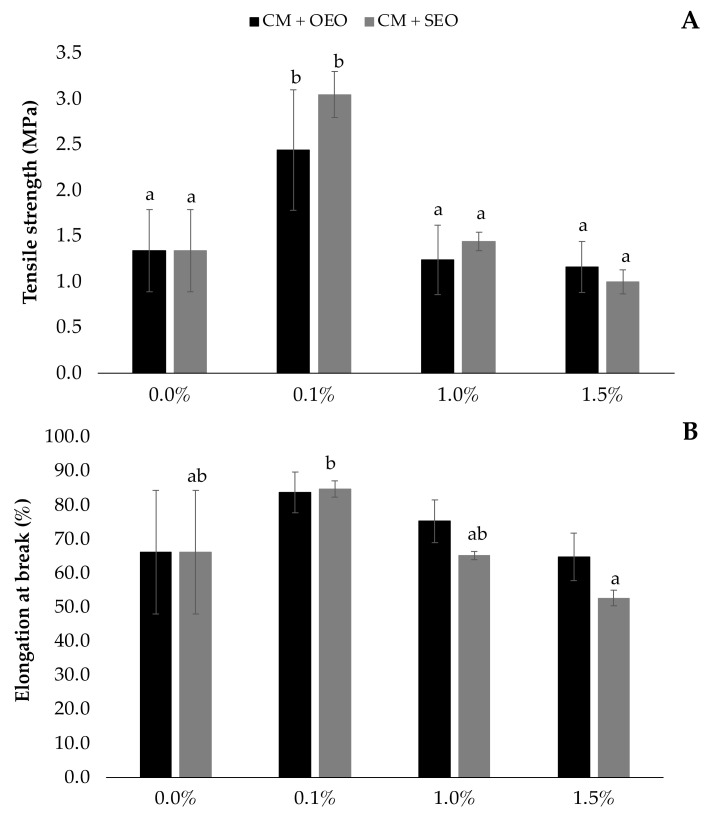
Effect of oregano (OEO) or savory (SEO) essential oil addition on tensile strength (**A**) and elongation at break (**B**) of chia mucilage films (CM, Muñoz-Tebar et al. [12]). ^a–b^ Different superscripts means significant differences (*p* < 0.05) due to EO concentration. Data without any superscripts mean that there were no significant differences (*p* > 0.05) either by the EO concentration effect (indicated with lower case letters) or by the EOs used in the formulation (indicated with capital letters).

The use of the two types of essential oils (oregano and savory) did not influence (*p* > 0.05) the mechanical response of the films at any concentration. However, increasing concentration of EOs from 1.0% to 1.5% *v*/*v* led to a significant decrease in TS and EB (Figure 2).

**Figure 3:**
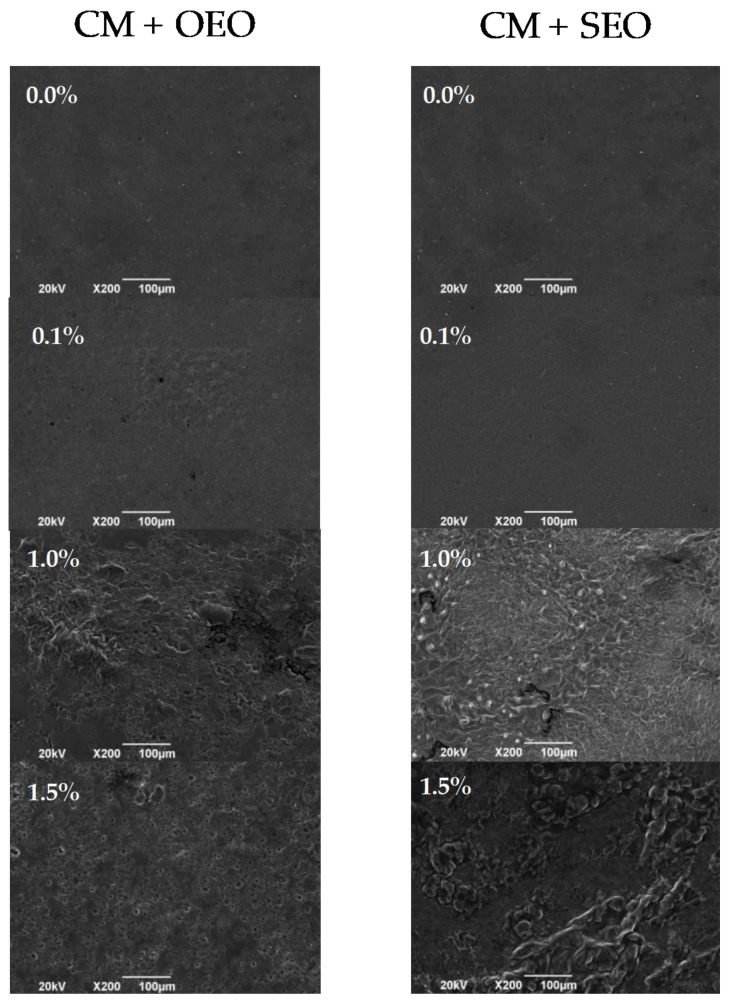
SEM micrographs of the film’s surface with different concentrations of oregano (OEO) and savory (SEO) essential oils.

The decrease in TS and EB of the films can be explained by the development of a heterogeneous surface and discontinuities. SEM micrographs (Figure 3) show that the films containing savory at the highest concentration had a more heterogeneous and rougher surface, which could explain why the reduction in mechanical properties was greater than in the films containing oregano essential oil (Figure 2). In addition, Atarés et al. [51] reported that moisture content plays an important role on the tensile properties of the films as an increase in moisture from 5% to 10% resulted in lower EB and TS values. Therefore, the results are consistent with the moisture increase in the films when the EOs were incorporated (Table 1).

A similar effect was observed by Pagno et al. [48], who incorporated oregano and thyme EOs into quinoa flour films obtaining reduced tensile strength values (from 3.0 to 1.2 MPa and from 2.8 to 1.8 MPa with oregano and thyme EOs, respectively), and by Hasheminya et al. [37], in which the incorporation of *Satureja khuzestanica* essential oil in kefiran–carboxymethyl cellulose films significantly reduced the elongation at break from 74.13% to 65.27%. Overall, the increase of the essential oil (both oregano and savory) concentrations resulted in softer, less resistant to break and less stretchable films.

### 3.5. Antifungal Capacity

One of the main targets of the incorporation of bioactive substances, such as essential oils, is to provide the films with biological properties. Table 3 shows the results of the antifungal activity of the different developed films against the five fungi strains studied (*A. flavus* CECT 2687, *A. puulaauensis* 1A05, *P. commune* 301, *P. crustosum* 1A01 and *P. verrucosum* CECT 2906), which were selected for being the most representative of pressed sheep cheeses [30]. Chia mucilage-based films and those formulated with 0.1% *v*/*v* essential oil (regardless of the type) did not show activity against any of the mold strains tested. From that concentration onwards, a significant increase was observed in the halos diameter and percentage of growth inhibition with increasing EO concentrations (regardless of the type).

However, it is noteworthy that the increase of the EO concentration in the films containing oregano had no significant effect on the inhibition of *P. verrucosum* CECT 2906, while films containing savory had no significant effect on the inhibition of *P. crustosum* 1A01 (Table 3). As it was expected, films containing 1.5% *v*/*v* of EO (regardless of the type) were the most effective against all the tested mold strains. Overall, films containing *O. vulgare* essential oil were more active than films incorporated with *S. montana* EO in all cases, except in *A. puulaauensis* 1A05 at 1.5% *v*/*v*, which was less active than savory films. Considering each of the tested mold species, Table 3 shows that the most resistant strains were *P. crustosum* 1A01 and *A. flavus* CECT 2687 (percentages of inhibition ranged from 29.38 to 47.90%), whereas the more sensitive were *A. puulaauensis* 1A05 (from 47.92 to 77.66% growth inhibition) against the two types of essential oil used in this study.

Noshirvani et al. [46] used cinnamon and ginger essential oils in chitosan–carboxymethyl cellulose films to inhibit the growth of *A. niger* achieving lower percentages of inhibition (24.9 and 43.5% for ginger and cinnamon, respectively) than those obtained in the present work against *A. puulaauensis* and *A. flavus*. Additionally, Souza et al. [52] incorporated cinnamon EO in cassava starch composite films to control the growth of *P. commune* and their percentage of inhibition was lower than the values of the present films containing oregano and savory EO (32.10 vs. 60.63–69.06% at 1.5% *v*/*v*, respectively). The high content of phenolic compounds, such as carvacrol and thymol present in *O. vulgare* and *S. montana* essential oils, are the responsible compounds for their strong antifungal activity [53]; the results confirm that the films would be effective in controlling fungal contamination and could be a suitable alternative to the use of plastic coatings (polyvinyl acetate) with synthetic substances, such as natamycin, which is used in coatings applied to cheese [54].

### 3.6. Scanning Electron Microscopy (SEM)

The SEM images from the surface of the control films and the films with different concentrations of oregano and savory essential oils are shown in Figure 3.

Chia mucilage-based films (without EOs) displayed a homogeneous and smooth surface with a slight presence of air bubbles as well as being compact without pores or cracks. Likewise, it was observed that the incorporation of essential oil at 0.1% *v*/*v* (regardless of the type) did not cause a significant effect on the microstructure of the films since they showed a homogeneous distribution of the essential oil droplets and a smooth surface with small irregularities and no porosity, similar to the pure CM film (Figure 3). On the contrary, the incorporation of essential oils at 1.0 and 1.5% *v*/*v* led to modifications in the microstructure of the films, with slightly different behaviors for both EOs.

Overall, films incorporated with both EOs presented surfaces without pores or cracks, which might indicate that the film solution had a stable emulsion system that was maintained during drying [37] and that both essential oils are compatible with the chia mucilage matrix. However, films incorporated with OEO showed a less rough and heterogeneous surface when compared to films embedded with SEO, which showed coalescence regions, suggesting a better incorporation of OEO withing the film matrix.

SEM micrographs exhibited that oil droplets were evenly entrapped in the continuous network of polysaccharide in films containing OEO at 1.5% *v*/*v*, while looser texture with sponge-like and oil aggregation was obtained in the films incorporated with SEO at the same concentration (Figure 3). In addition, oil droplets were not completely spherical, which is common in oil-in-water type emulsions [42].

## 4. Conclusions

Chia mucilage films incorporated with different concentrations of oregano and savory essential oils were successfully prepared by casting technique using glycerol as a plasticizer and Tween 20 as an emulsifier and surfactant. This study suggested that the incorporation of oregano and savory essential oils into biodegradable films had a significant influence on physical, transmittance, antifungal and mechanical properties of the subsequent chia mucilage EO films. The addition of increasing concentrations of EO (regardless of the type) led to an increase in moisture content, solubility and thickness. No significant changes were observed due to the EOs’ addition in physical and optical properties, with the exception of ∆E that was significantly higher in the films containing SEO. The films (OEO and SEO) exhibited good protection against UV radiation and films containing savory EO were better barriers against visible light proving their suitability as films for foods with high lipid contents to minimize oxidative reactions. Despite the fact that incorporation of increasing EO concentrations significantly modify the mechanical properties of the films, they still present good elongation at break values, being malleable and having a high degree of extensibility and deformation. Moreover, chia mucilage EO films containing oregano and savory exhibited high antifungal effect in in vitro tests against all the tested mold strains. The film’s microstructure analyzed by SEM revealed that the incorporation of EOs led to a more heterogeneous and rougher film’s surface without porosity and cracks, and the use of oregano resulted in a better incorporation and distribution of the essential oil in the polymeric matrix.

The research results are encouraging because they demonstrate that the films made with mucilage extracted from defatted chia seeds and incorporated with *O. vulgare* and *S. montana* essential oils have appreciable potential to be used as a natural and antimicrobial packaging to control mold contamination and extend the shelf life of foods. However, further in vivo studies are required to better understand the effects of these chia by-product films on the foods as an alternative to the use of plastic films that are normally used in the agri-food industry.

## Figures and Tables

**Table 1:** Effect of oregano (OEO) or savory (SEO) essential oil addition in the physical properties of the chia mucilage films (CM) (mean ± sd).

Parameter	Concentration (%)	Films	
		CM + OEO	CM + SEO
Moisture (%)	0.0 ^1^	22.44 ± 1.19 ^a^	22.44 ± 1.19 ^a^
	0.1	24.80 ± 0.95 ^ab^	25.73 ± 0.52 ^ab^
	1.0	25.31 ± 0.21 ^ab^	27.60 ± 1.72 ^bc^
	1.5	27.69 ± 2.05 ^b^	30.83 ± 2.76 ^c^
Water solubility (%)	0.0 ^1^	64.45 ± 3.90 ^a^	64.45 ± 3.90 ^a^
	0.1	64.98 ± 0.57 ^a^	65.39 ± 1.00 ^a^
	1.0	67.53 ± 0.30 ^a^	68.64 ± 0.21 ^a^
	1.5	75.11 ± 0.66 ^b^	75.60 ± 0.30 ^b^
Thickness (mm)	0.0 ^1^	0.123 ± 0.010 ^b^	0.123 ± 0.010 ^b^
	0.1	0.075 ± 0.007 ^a^	0.082 ± 0.001 ^a^
	1.0	0.085 ± 0.001 ^a^	0.090 ± 0.004 ^a^
	1.5	0.092 ± 0.002 ^a^	0.096 ± 0.004 ^a^
WVP ^2^ × 10^−10^ (g/s m Pa)	0.0 ^1^	0.58 ± 0.03 ^b^	0.58 ± 0.03 ^b^
	0.1	0.14 ± 0.03 ^a^	0.14 ± 0.02 ^a^
	1.0	0.16 ± 0.03 ^a^	0.18 ± 0.03 ^a^
	1.5	0.17 ± 0.03 ^a^	0.20 ± 0.01 ^a^

^1^ Values obtained in a previous work (Muñoz-Tebar et al. [12]); ^2^ WVP: water vapor permeability; ^a–c^ Different superscripts between row means significant differences (*p* < 0.05) due to EO concentration. Data without any superscripts mean that there were no significant differences (*p* > 0.05) either by the EO concentration effect (indicated with lower case letters) or by the EOs used in the formulation (indicated with capital letters).

**Table 2:** Effect of oregano (OEO) or savory (SEO) essential oil addition in the optical properties of the chia mucilage films (CM) (mean ± sd).

Parameter	Concentration (%)	Films	
		CM + OEO	CM + SEO
L*	0.0^1^	56.42 ± 4.15	56.42 ± 4.15
	0.1	53.03 ± 0.96	51.27 ± 1.90
	1.0	50.64 ± 2.33	53.27 ± 1.28
	1.5	50.18 ± 1.45	50.32 ± 1.57
a*	0.0 ^1^	5.45 ± 0.32 ^a^	5.45 ± 0.32 ^a^
	0.1	6.35 ± 0.70 ^ab^	6.93 ± 1.04 ^b^
	1.0	7.90 ± 1.63 ^bc^	6.55 ± 0.54 ^b^
	1.5	8.66 ± 0.74 ^c^	8.74 ± 0.79 ^c^
b*	0.0 ^1^	37.39 ± 4.16	37.39 ± 4.16
	0.1	37.98 ± 0.41	38.13 ± 0.34
	1.0	36.57 ± 1.58	36.38 ± 1.17
	1.5	36.31 ± 1.44	36.83 ± 0.61
∆E	0.0 ^1^	54.40 ± 5.71	54.40 ± 5.71
	0.1	57.45 ± 0.92 ^A^	64.33 ± 1.55 ^B^
	1.0	58.68 ± 2.37 ^A^	65.14 ± 0.84 ^B^
	1.5	59.01 ± 1.06 ^A^	63.00 ± 1.50 ^B^
Opacity (%)	0.0 ^1^	26.87 ± 0.75 ^a^	26.87 ± 0.75 ^a^
	0.1	28.80 ± 0.11 ^ab^	28.09 ± 0.50 ^ab^
	1.0	29.73 ± 3.18 ^ab^	29.60 ± 0.75 ^ab^
	1.5	31.48 ± 0.32 ^b^	30.86 ± 2.41 ^b^

^1^ Values obtained in a previous work (Muñoz-Tebar et al. [12]); ^a–c^ Different superscripts between row means significant differences (*p* < 0.05) due to EO concentration. ^A,B^ Different superscripts between columns means significant differences (*p* < 0.05) due to the essential oils used in the film formulation. Data without any superscripts mean that there were no significant differences (*p* > 0.05) either by the EO concentration effect (indicated with lower case letters) or by the EOs used in the formulation (indicated with capital letters).

**Table 3:** Effect of oregano (OEO) or savory (SEO) essential oil addition in the antifungal activity (halos and percentage of inhibition) of the chia mucilage films (CM) (mean ± sd).

Mold	Parameter	Concentration (%)	Films	
			CM + OEO	CM + SEO
*Aspergillus flavus* CECT 2687	Halo (mm)	0.0 ^1^	nd ^2^	nd ^2^
		0.1	nd ^2^	nd ^2^
		1	31.35 ± 2.83 ^a^	28.94 ± 1.19 ^a^
		1.5	47.90 ± 4.49 ^b^	41.62 ± 1.70 ^b^
	% inhibition	0.0 ^1^	nd ^2^	nd ^2^
		0.1	nd ^2^	nd ^2^
		1	34.83 ± 3.15 ^a^	32.15 ± 1.32 ^a^
		1.5	53.22 ± 4.99 ^b^	46.25 ± 1.89 ^b^
*Aspergillus puulauensis* 1AO5	Halo (mm)	0.0 ^1^	nd ^2^	nd ^2^
		0.1	nd ^2^	nd ^2^
		1	51.56 ± 0.95 ^aA^	43.13 ± 1.41 ^aA^
		1.5	64.86 ± 0.06 ^bA^	69.89 ± 0.82 ^bB^
	% inhibition	0.0 ^1^	nd ^2^	nd ^2^
		0.1	nd ^2^	nd ^2^
		1	57.29 ± 1.06 ^aB^	47.92 ± 1.57 ^aA^
		1.5	72.07 ± 0.06 ^bA^	77.66 ± 0.91 ^bB^
*Penicillium commune* 301	Halo (mm)	0.0^1^	nd ^2^	nd ^2^
		0.1	nd ^2^	nd ^2^
		1	39.28 ± 0.26 ^aB^	34.47 ± 0.23 ^aA^
		1.5	62.16 ± 2.34 ^b^	54.56 ± 5.20 ^b^
	% inhibition	0.0 ^1^	nd ^2^	nd ^2^
		0.1	nd ^2^	nd ^2^
		1	43.65 ± 0.29 ^aB^	38.30 ± 0.25 ^aA^
		1.5	69.06 ± 2.60 ^b^	60.63 ± 5.78 ^b^
*Penicillium crustosum* 1AO1	Halo (mm)	0.0 ^1^	nd ^2^	nd ^2^
		0.1	nd ^2^	nd ^2^
		1	28.95 ± 0.41 ^a^	26.44 ± 1.72
		1.5	39.45 ± 2.40 ^b^	38.01 ± 3.91
	% inhibition	0.0 ^1^	nd ^2^	nd ^2^
		0.1	nd ^2^	nd ^2^
		1	32.16 ± 0.46 ^a^	29.38 ± 1.91
		1.5	43.84 ± 2.67 ^b^	42.23 ± 4.34
*Penicillium verrucosum* CECT 2906	Halo (mm)	0.0 ^1^	nd ^2^	nd ^2^
		0.1	nd ^2^	nd ^2^
		1	43.19 ± 4.08	33.53 ± 3.97 ^a^
		1.5	56.43 ± 6.74	50.38 ± 1.99 ^b^
	% inhibition	0.0 ^1^	nd ^2^	nd ^2^
		0.1	nd ^2^	nd ^2^
		1	47.99 ± 4.53	37.25 ± 4.41 ^a^
		1.5	62.70 ± 7.49	55.98 ± 2.21 ^b^

^1^ Values obtained in a previous study (Muñoz-Tebar et al. [12]); ^2^ nd: no inhibition detected; ^a,b^ Different superscripts between row means significant differences (*p* < 0.05) due to EO concentration. ^A,B^ Different superscripts between columns means significant differences (*p* < 0.05) due to the essential oils used in the film formulation. Data without any superscripts mean that there were no significant differences (*p* > 0.05) either by EOs concentration effect (indicated with lower case letters) or by the EOs used in the formulation (indicated with capital letters).

## Data Availability

The data presented in this study are available on request from the corresponding author.

## Supplementary Materials

The following supporting information can be downloaded at: https://www.mdpi.com/article/10.3390/membranes12020213/s1, Table S1: Effect of oregano (OEO) or savory (SEO) essential oil in the light transmittance (%) of chia mucilage films (CM).
