# Supplementary material for: Chia Seed Mucilage Edible Films with Origanum vulgare and Satureja montana Essential Oils: Characterization and Antifungal Properties
Source: Membranes (Basel). 2022 Feb 11;12(2):213. doi: 10.3390/membranes12020213 (PMC8875529; doi:10.3390/membranes12020213)
Supplement: Supplementary file 1 [file membranes-12-00213-s001.zip › membranes-1500894-supplementary.pdf]

Supplementary Materials: Chia Seed Mucilage Edible Films with *Origanum vulgare* and *Satureja montana* Essential Oils: Characterization and Antifungal Properties

Nuria Muñoz-Tébar [1], Manuel Carmona [1], Gonzalo Ortiz de Elguea-Culebras [2], Ana Molina [1] and María Isabel Berruga [1,*]

[1] Food Quality Research Group, Institute for Regional Development (IDR), Universidad de Castilla-La Mancha, 02071 Albacete, Spain; nuria.munoz@uclm.es (N.M.-T.); manuel.carmona@uclm.es (M.C.); ana.molina@uclm.es (A.M.)

[2] Centro de Investigación Agroforestal de Albaladejito (IRIAF-JCCM), Carretera Toledo-Cuenca km 174, 16194 Cuenca, Spain; gonzaloo@jccm.es

* Correspondence: mariaisabel.berruga@uclm.es; Tel.: +34-599200 (ext. 2615)

Table S1. Effect of oregano (OEO) or savory (SEO) essential oil in the light transmittance (%) of chia mucilage films (CM).

Films	Wavelength (nm)								
	500 [2]	550	600	650	700	750	800	850	900
CM + OEO-0.0%[1]	21.75 ± 7.41	31. 98 ± 7.75^{ab}	40.19 ± 7.40^{b}	45.65 ± 6.06^{b}	51.64 ± 6.41^{b}	58.12 ± 5.67^{b}	62.99 ± 5.09^{b}	66.27 ± 4.76 ^{b}	68.65 ± 4.52 ^{b}
CM + OEO-0.1%	12.28 ± 5.20^{A}	19.70 ± 5.80^{aA}	26.31 ± 5.68^{aA}	31.61 ± 5.30^{aA}	37.25 ± 4.64^{aA}	44.16 ± 3.73^{aA}	49.48 ± 2.94^{aA}	53.47 ± 2.27 ^{aA}	56.51 ± 1.81 ^{aA}
CM + OEO-1.0%	22.04 ± 2.68^{B}	32.70 ± 2.65^{abBC}	40.99 ± 2.67^{bCD}	47.05 ± 2.66^{bCD}	52.90 ± 2.89^{bCD}	60.04 ± 2.36^{bD}	64.84 ± 2.23^{bD}	68.19 ± 2.08 ^{bD}	70.39 ± 1.90 ^{bC}
CM + OEO-1.5%	23.25 ± 3.52^{B}	35.78 ± 3.76^{bC}	45.54 ± 3.47^{bD}	52.58 ± 3.18^{bD}	59.39 ± 2.67^{bD}	67.60 ± 1.95^{bE}	73.17 ± 1.39^{cE}	76.86 ± 0.95 ^{cE}	79.19 ± 0.83 ^{cD}
CM + SEO-0.0%[1]	21.75 ± 7.41	31. 98 ± 7.75	40.19 ± 7.40	45.65 ± 6.06	51.64 ± 6.41	58.12 ± 5.67	62.99 ± 5.09^{b}	66.27 ± 4.76 ^{b}	68.65 ± 4.52 ^{b}
CM + SEO-0.1%	21.29 ± 1.99^{B}	31.09 ± 1.57^{BC}	38.89 ± 1.22^{BCD}	44.46 ± 0.90^{BC}	50.02 ± 0.52^{BC}	56.85 ± 0.74^{CD}	61.84 ± 1.17^{abCD}	65.40 ± 1.48 ^{abCD}	68.02 ± 1.71 ^{bC}
CM + SEO-1.0%	16.29 ± 0.95^{AB}	26.18 ± 1.25^{AB}	34.04 ± 1.39^{ABC}	39.68 ± 1.34^{BC}	45.33 ± 1.29^{B}	52.35 ± 1.15^{BC}	57.44 ± 0.95^{abBC}	60.98 ± 0.75 ^{abBC}	63.51 ± 0.63 ^{abB}
CM + SEO-1.5%	15.81 ± 1.38^{AB}	25.13 ± 1.49^{AB}	32.40 ± 1.20^{AB}	37.75 ± 0.97^{AB}	43.07 ± 0.87^{AB}	49.55 ± 0.80^{AB}	54.31 ± 1.29^{aAB}	57.65 ± 1.72 ^{aAB}	59.93 ± 2.04 ^{aAB}

[1] Values obtained in a previous work (Muñoz-Tebar et al. [12])

[2] Not significant differences from 200 to 450 nm.

$^{a-c}$ Different superscripts between row means significant differences ($p < 0.05$) due to EOs concentration.

$^{A-B}$ Different superscripts between columns means significant differences ($p < 0.05$) due to the essential oils used in the film formulation.

Data without any superscripts means that there were no significant differences ($p > 0.05$) either by EOs concentration effect (indicated with lower case letters) or by the EOs used in the formulation (indicated with capital letters).
